# Household expenditure on leprosy outpatient services in the Indian health system: A comparative study

**DOI:** 10.1371/journal.pntd.0006181

**Published:** 2018-01-04

**Authors:** Anuj Tiwari, Pramilesh Suryawanshi, Akash Raikwar, Mohammad Arif, Jan Hendrik Richardus

**Affiliations:** 1 Department of Public Health, Erasmus MC, University Medical Center Rotterdam, Rotterdam, The Netherlands; 2 Netherlands Leprosy Relief, India Branch, New Delhi, India; London School of Hygiene and Tropical Medicine, UNITED KINGDOM

## Abstract

**Background:**

Leprosy is a major public health problem in many low and middle income countries, especially in India, and contributes considerably to the global burden of the disease. Leprosy and poverty are closely associated, and therefore the economic burden of leprosy is a concern. However, evidence on patient’s expenditure is scarce. In this study, we estimate the expenditure in primary care (outpatient) by leprosy households in two different public health settings.

**Methodology/Principal findings:**

We performed a cross-sectional study, comparing the Union Territory of Dadra and Nagar Haveli with the Umbergaon block of Valsad, Gujrat, India. A household (HH) survey was conducted between May and October, 2016. We calculated direct and indirect expenditure by zero inflated negative binomial and negative binomial regression. The sampled households were comparable on socioeconomic indicators. The mean direct expenditure was USD 6.5 (95% CI: 2.4–17.9) in Dadra and Nagar Haveli and USD 5.4 (95% CI: 3.8–7.9) per visit in Umbergaon. The mean indirect expenditure was USD 8.7 (95% CI: 7.2–10.6) in Dadra and Nagar Haveli and USD 12.4 (95% CI: 7.0–21.9) in Umbergaon. The age of the leprosy patients and type of health facilities were the major predictors of total expenditure on leprosy primary care. The higher the age, the higher the expenditure at both sites. The private facilities are more expensive than the government facilities at both sites. If the public health system is enhanced, government facilities are the first preference for patients.

**Conclusions/Significance:**

An enhanced public health system reduces the patient’s expenditure and improves the health seeking behaviour. We recommend investing in health system strengthening to reduce the economic burden of leprosy.

## Author summary

Leprosy leads to low quality of life even after cure. The anaesthetic hands and feet leading to ulcers and deformities, stigma and poor mental health are just a few challenges. After declaration of leprosy elimination at global level, the research activities reduced significantly, and the health economics aspect was not an exception. The knowledge on economic burden of a disease helps in prioritization, policy making and advocacy. Our study is a step towards quantifying the economic burden of leprosy. Currently the aim is to eliminate leprosy at national level, therefore the countries need more information to plan high impact activities. Moreover, the patient’s perspective is important as they are the end-point recipients. Our study explores the patient’s financial burden due to leprosy (outpatient services), which is a significant indicator of a public health program’s success. If invested properly, the public health system has potential to reduce the economic burden of public health diseases. Our study is an attempt to link the patient’s perspective with the health system performance. This will help to encourage health systems strengthening.

## Introduction

Leprosy is caused by *Mycobacterium leprae*, affecting the peripheral skin, nerve and nasal mucosa [1]. The adverse impact of leprosy on human lives is serious due to nerve function impairment and disabilities. Moreover, the early manifestation of disability in the form of sensory loss of hands or feet, often fails to seize attention of clinicians and patients, resulting into detection delay and further transmission of *M*. *leprae* [2, 3]. Therefore, the annual new case detection rate (NCDR) of leprosy is stagnant since many years [4]. The expectation to permanently eradicate leprosy, also referred as zero transmission [5] is now reflected into new WHO targets i.e. zero grade 2 disabilities among children, and new cases with grade 2 disability <1 case/million population [6]. However, the targets are difficult to achieve in the near future [7, 8], which means that leprosy will keep on imposing burden in many endemic countries.

Leprosy and poor socioeconomic status are in a vicious cycle, characterized by inequality [9–11], poor education [12], poverty [13, 14], stigma, etc. [15, 16]. A broad spectrum of evidence confirms the strength of the relationship between leprosy and poverty [17–21]. Evidence from Bangladesh shows that leprosy affected households have a poor nutritional level due to lower food expenditure per capita and household food stocks. This in fact increases the risk of acquiring leprosy in healthy household members [22]. Another study revealed that “people affected by leprosy are less likely to be stigmatized because of leprosy impairments than for their incapacity to contribute to family/community finances” [23]. Furthermore, leprosy incidence is high in the productive age group, resulting in long term financial loss [17]. Therefore, we suspect that the economic burden of leprosy is higher than perceived so far.

Household expenditure represents the patient’s perspective and is critical in estimating the economic burden. It is now routinely done across diseases [24], revealing underlying expenditure like income loss, which can sometimes be significant. Unfortunately, the cost evidence in leprosy is limited [25]. A literature search on PubMed using a broad search builder with ‘leprosy’ as MeSH term and ‘economics’ as sub-MeSH heading (year 2001 onwards), resulted in 51 records. Only 6 records presented some cost estimates: three studies focused on a particular event (ENL reaction and ulceration) in hospital settings [26–28]; two cost-effectiveness analysis (CEA) studies on provider’s perspective [29, 30]; and one study on human resource cost of a project [31]. No study was found exclusively on primary care in a general public health setting, covering the patient’s perspective.

Leprosy is a chronic infectious disease with long treatment duration, therefore needs long term care and support, mainly in an outpatient setting. Therefore, the primary objective of our study is to estimate the expenditure in primary (outpatient) care incurred by leprosy patients in two different health system settings in India. The secondary objective is to compare the effect of the health systems on consumer behaviour and practices. The results will help in understanding the economic burden of leprosy in primary care, and eventually contribute in building an investment case for leprosy elimination [25].

## Methods

### Ethics statement

The study was conducted under the Leprosy Post Exposure Prophylaxis (LPEP) program, approved in India by the Institutional Human Ethics Committees of the National Institute of Epidemiology (NIE/IHEC201407-01). Written informed consent was received from the respondents and necessary permission was taken from the concerned departments.

### Background of LPEP in India

India contributes almost 60% to the global leprosy burden [4]. The LPEP program was launched in March 2015 in the Union Territory of Dadra and Nagar Haveli (DNH), located on the western coast of India. The program aims to assess impact and feasibility of contact tracing and administration of single dose of rifampicin (SDR) to asymptomatic contacts of leprosy cases. LPEP is implemented by the National Leprosy Elimination Program (NLEP) of India [32].

### Study design

The study followed a cross-sectional design, where a cohort from the Union Territory of DNH was compared with a cohort from Umbergaon block of Valsad district, Gujarat, India. A union territory is an administrative division, ruled directly by the federal government, whereas a block is the smallest administrative unit under a district. The cohorts were leprosy cases detected between April 2015 and March, 2016. A sample of 120 participants from each group was selected randomly from the annual leprosy case detection list. In the financial year of 2015–16, DNH reported 425 and Umbergaon reported 287 cases.

### Study sites

DNH and Umbergaon share boundaries and are comparable with regard to demographic, epidemiological, and socioeconomic indicators (Table 1), but not to public health facilities due to the different governmental arrangement (see below).

**Table 1:** Comparison of Dadra and Nagar Haveli and Umbergaon with regard to demography, epidemiology, socioeconomics factors, and public health facilities.

Indicators	DNH	Umbergaon
*Demographic & Socioeconomic indicators (Census 2011)*		
Number of households (HH)	76,121	54,814
Population	343,709	261,204
Rural population	53.27%	68.74%
Females (per 1000 males)	774	933
Literacy	76.24%	69.53%
Schedule tribes^#^	51.95%	51.32%
Total working population	45.73%	40.40%
*Epidemiology (2015–16)*		
Leprosy screened population	388,613	371,731
New cases detected	425	287
NCDR* (per 100,000 per year)	109.36	77.21
New child cases (age < 15 years)	23.29%	16.03%
New female cases	57.88%	61.67%
Prevalence rate (per 10,000 per year)	6.77	3.81
Grade II disability in new cases	3.3%	2.44%
PB/MB** ratio	2.76	3.15
*Public Health Infrastructure (2015–16)*		
Area (sq. km)	491	343
Primary health centres (PHC)	15	10
Sub-centres	50	64
Average population screened for leprosy by health centre	25,907	37,173

^#^The Scheduled Castes (SCs) and Scheduled Tribes (STs) are various officially designated groups of historically disadvantaged indigenous people in India.

* NCDR: new case detection rate

** PB: Paucibacillary; MB: Multibacillary

Both study sites are mainly tribal areas, but there is a remarkable difference in the public health system of both sites. The public health system in DNH is enhanced because it falls directly under the federal government by bypassing provincial bureaucracy, and receives a higher health budget per capita [33–35] than the provinces. In comparison to DNH, Umbergaon has more PHCs per population covered; the average population screened for leprosy by a Primary Health Center (PHC) in Umbergaon was 43% more than DNH PHC (Table 1). The actual screening (active and passive) coverage was reported to be very high in both sites, approximating the total population of these areas. In the year 2015–16, the leprosy program performed two active case detection surveys in both sites. Currently both sites fall under the Leprosy Case Detection Campaign (LCDC), which was launched in early 2016 under the NLEP [36]. Furthermore, the population screened by Umbergaon PHCs is far more than the public health norms for tribal PHCs, i.e. 86% more in Umbergaon and 26% in DNH [37]. Typically, a PHC should cover a population of 20,000 in hilly, tribal, or difficult areas and 30,000 populations in plain areas [37]. Both sites provide free of charge leprosy outpatient department (OPD) services at all public health facilities, but the health systems vary with regard to infrastructure, availability, accessibility, and quality of services.

### Data collection and analysis

A household survey was conducted between June and October, 2016 by means of a structured questionnaire. The data were collected by two experienced staff members, post-graduates in public health. The patient, or head of the household, or most knowledgeable person in the household was asked to report on patient demographics, HH socioeconomic status, accessibility of health services, treatment seeking history and OPD expenditure. Respondents were asked to report on the last three OPD visits, either in a public or private facility, in the last 6 months. The database was created in Excel. The analysis included only those patients who mentioned at least 1 OPD visit out of 3.

The costs were categorized as direct and indirect expenditure. The direct part included the expenditure on consultation, investigations and medicines & supplies. The indirect part constituted expenditure on transport, food, and days lost during illness of the patient and attendant. We calculated the transportation expenditure by multiplying to-and-fro distance from house to the nearest health facility, using the government transportation rate [38]. The wage loss was analysed by means of the human capital approach [39]. The wage losses for patients and attendants per illness episode were calculated by using government minimum wage rates [40]. There were 20 (8%) patients who paid at least 1 OPD visit, but failed to report any loss of productive days. For these, we imputed half a day wage loss per visit under the assumption that at least half a day (4 hours) is required to travel and avail services for each illness episode. But attendant’s productive day loss could be zero, as not all patients required attendants. We reported separately the days lost by child patients (age < 16 years) as ‘school days lost’, but while calculating indirect expenditure, all patients and attendants were assumed to be 16 years and older. The results are presented in US dollars (USD) using the conversion rate of INR 67 for 1 dollar for the year 2016 [41]. The analyzed expenditure was exclusively of outpatient services.

### Data modelling

In order to answer our objectives, i.e. expenditure and patient’s health seeking behaviour differences in DNH and Umbergaon, we used an integrated analytical approach. The data distribution was evaluated by observing normality plots. The distribution of the direct expenditure variables were not normally distributed due to abundance of zeros and highly skewed for non-zero values, which is common in cost data [42]. The indirect expenditure variables were skewed, but not zero inflated. We compared four different distribution models, i.e. Poisson, negative binomial, zero inflated Poisson, and zero inflated negative binomial distribution [43]. The ‘zero inflated negative binomial regression’ was selected for direct expenditure variables, and ‘negative binomial regression’ for indirect and total expenditure variables. We estimated the mean expenditure for each variable, followed by association measurement between expenditure and patient’s household characteristics. Only significant (p <0.05) variables were modelled together for multivariate regression analysis (Generalized Linear Model). The magnitude of total expenditure was compared against the individual’s monthly income. The total per visit expenditure was defined catastrophic for an individual, if it exceeded 10% of the quarterly income [44, 45]. We assumed that at least one visit to the health centre in a quarter is necessary for regular check-up of leprosy. However as per NLEP norms, patients should visit the health center every month, which rarely happens. In practice, monthly MDT is delivered by staff at the patient’s doorstep and health facility visits occur only during severe illnesses to avoid any wage loss.

## Results

A total of 240 patient households (120 in each group) were approached to capture their characteristics and OPD visit details in the last 6 months. The area-wise household characteristics are summarized in Table 2. The mean age (DNH: 25, Umbergaon: 24) showed a young and comparable population in both sites. The average monthly income (DNH: USD 81, Umbergaon: USD 97), expenditure (DNH: USD 73, Umbergaon: USD 83) and saving (DNH: USD 1 Umbergaon: USD 1) per earning member showed a poor economic status in both sites. The respondents differed prominently on characteristics such as distance to the nearest health facility, type of housing, OPD frequency and type of facility visited. Paucibacillary (PB) leprosy was more prevalent in both sites than multibacillary (MB) leprosy. Collectively in the three visits, 69% of the respondents in Umbergaon and 14% of the respondents in DNH had not paid any visit, and were therefore dropped for further analysis.

**Table 2:** Socioeconomic characteristics of patient households in DNH and Umbergaon.

	DNH (N = 120)		Umbergaon (N = 120)		p
	Mean (USD)	95% CI	Mean (USD)	95% CI	
Age (years)	24.7	22.0–27.7	23.6	17.9–31.1	0.58
HH size	6.0	5.6–6.4	5.4	4.7–6.3	0.03
Number of earning members	1.5	1.3–1.7	1.6	1.1–2.3	0.41
Monthly income per earning member in HH in INR	5,456 (81)	5,144–5,787	6,503 (97)	5,642–7,495	0.00
Monthly expenditure per earning member in HH in INR	4,890 (73)	4,566–5,238	5,591 (83)	4,736–6,601	0.01
Monthly savings per earning member in HH in INR	74 (1)	41–133	87 (1)	47–161	0.71
Distance of nearest health facility (km)	5.1	4.6–5.6	9	8.0–9.9	0.00
	**N**	**%**	**N**	**%**	**p **
Sex: Female	73	60.8	70	58.3	0.69
Occupation: Not Earning*	87	72.5	67	55.8	0.01
Leprosy type: PB	104	86.7	92	76.7	0.05
Type of housing: Concrete predominant**	95	79.2	68	56.7	0.00
OPD frequency (Max 3. duration last 6 months)					
0	17	14.2	83	69.2	0.00
1	77	64.2	25	20.8	
2	24	20.0	11	9.2	
3	2	1.7	1	0.8	
Type of OPD facility (last 3 visits in 6 months)					
No visit	17	14.2	83	69.2	
Only government	97	80.8	14	11.7	0.00
Both	4	3.3	5	4.2	
Only private	2	1.7	18	15.0	

*Not earning in comparison to earning, includes unemployed, children, housewives

** In comparison to mud predominant houses

The three visits expenditure was aggregated to obtain an average per visit. The details of direct and indirect expenditure are shown in Table 3. DNH and Umbergaon were comparable on demographic and socioeconomic parameters, however, they statistically significantly differed with regard to health seeking behaviour. As a behaviour, OPD visit frequency is higher, and a government facility is more preferred in DNH as compared to Umbergaon.

**Table 3:** Direct and indirect expenditure in INR by leprosy patients on outpatient care in DNH and Umbergaon.

	DNH					Umbergaon					p
	n reported	% N = 0	*Pr N = 0	Mean (USD)	95% CI	n reported	% N = 0	*Pr N = 0	Mean (USD)	95% CI	
OPD direct expenditure per visit**											
Consultation	103	89	0.90	78 (1.2)	36–171	37	38	0.36	107 (1.6)	81–143	0.22
Medicines & supplies	103	91	0.89	478 (7.1)	167–1394	37	33	0.38	265 (4)	185–380	0.10
Total medical direct exp.	103	89	0.88	433 (6.5)	158–1200	37	33	0.35	365 (5.4)	252–528	0.60
Transport (non-medical direct)	103	0		54 (0.8)	45–66	37	0		94 (1.4)	53–166	**0.005**
OPD indirect expenditure per visit (wage loss per illness episode)**											
Patient's wage loss (age>15)	77	0		264 (3.9)	211–330	25	0		306 (4.6)	156–601	0.53
School days lost (Age<16)	26	0		2	1–3	12	25		3	1–10	0.38
Patient's wage loss (assumed all adults)	103	0		346 (5.2)	285–420	37	0		489 (7.3)	277–864	0.07
Attendant’s wage loss	103	32		183 (2.7)	151–223	37	19		246 (3.7)	139–436	0.13
Indirect exp.+ Transport (assumed all adult)	103	0		583 (8.7)	481–708	37	0		829 (12.4)	469–1464	0.07
Total (direct+ indirect) exp. (assumed all adults)	103	0		634 (9.5)	523–769	37	0		1075 (16)	609–1901	**0.006**

* Pr N = 0: predicted probability of 0 expenditure

**Medical direct expenditure (exp.) estimates are derived by zero inflated negative binomial regression.

Non-medical direct, Indirect and Total exp. estimates are derived by negative binomial regression.

Investigations and food were reported negligible, therefore, not included in the table.

All the presented expenditure estimates are per visit. The mean consultation fee in DNH and Umbergaon was comparable (DNH: USD 1.2, Umbergaon: USD 1.6). The mean expenditure on medicines and supplies (USD 7) was 80% higher in DNH than Umbergaon (USD 4). Only 2 respondents reported investigation expenditure in Umbergaon and none in DNH. Only 1 respondent in Umbergaon and 2 respondents in DNH reported expenditure on food. The mean medical direct expenditure per visit (DNH: USD 6.5, Umbergaon: USD 5.4) was not statistically significantly different between the sites. In indirect expenditure, the mean wage loss for patients was the highest item (DNH: USD 5.2, Umbergaon: USD 7.3), followed by attendant wage loss (DNH: USD 2.7, Umbergaon: USD 3.7). Transportation expenditure (DNH: USD 0.8, Umbergaon: USD 1.4) differed significantly (p ≤ 0.01) in the two groups.

The details on association of expenditures with patient’s household characteristics are shown in Table 4. The proportion of patients with catastrophic expenditure in DNH was 88% less than in Umbergaon. If catastrophic expenditure occurred, then direct expenditure rose three-fold in DNH and two-fold in Umbergaon, (DNH: coef. 2.92, 95% CI: 1.86–3.98; Umbergaon: coef. 1.00, 95% CI: 0.23–1.77). In DNH, the direct expenditure decreased statistically significantly more than two-fold (coef. -2.49, 95% CI: -3.74 to -1.24) with the increase in age groups, whereas a decrease in indirect expenditure against age was not statistically significant (coef. -0.40, 95% CI: -0.92 to 0.12). Umbergaon’s indirect expenditure decreased statistically significantly more than half (coef. -0.79, 95% CI: -1.49 to -0.09) among patients who visited both (government and private) facilities in comparison to those who visited only private facilities. For total expenditure, age and type of facility remained statistically significant factors, whereas catastrophic expenditure remained statistically significant only in DNH. Therefore these factors were considered for the next level of analysis, i.e. multivariate regression.

**Table 4:** Socioeconomic factors associated with expenditures by leprosy patients on outpatient services in DNH and Umbergaon (bivariate analyses).

		DNH									Umbergaon								
		Direct			Indirect			Total			Direct			Indirect			Total		
		Coef.	95% CI	P	Coef.	95% CI	p	Coef.	95% CI	P	Coef.	95% CI	p	Coef.	95% CI	p	Coef.	95% CI	p
Age	< = 18	1. (Ref)			1. (Ref)			1. (Ref)			1. (Ref)			1. (Ref)			1. (Ref)		
	19–35	-2.29	-3.66 -0.93	**.001**	-0.56	-1.00 -0.12	**.01**	-0.68	-1.12 -0.25	**.002**	0.01	-0.52 0.54	.97	-0.75	-1.52 0.02	.06	-0.87	-1.64 -0.10	**.03**
	> = 36	-2.49	-3.74 -1.24	**.000**	-0.40	-0.92 0.12	.13	-0.52	-1.04 0.00	**.048**	-0.23	-0.90 0.45	.51	-0.53	-1.33 0.28	.20	-0.47	-1.27 0.33	.25
Sex	Male	1. (Ref)			1. (Ref)			1. (Ref)			1. (Ref)			1. (Ref)			1. (Ref)		
	Female	-0.43	-2.02 1.15	.59	-0.11	-0.50 0.29	.60	-0.14	-0.54 0.25	.48	-0.32	-0.80 0.14	.17	-0.26	-0.97 0.44	.47	-0.39	-1.09 0.32	.28
Occupation	Earning	1. (Ref)			1. (Ref)			1. (Ref)			1. (Ref)			1. (Ref)			1. (Ref)		
	Not Earning	2.09	0.13 4.05	**.04**	0.29	-0.14 0.71	.18	0.38	-0.04 0.81	.08	0.39	-0.09 0.89	.11	0.13	-0.60 0.85	.74	0.13	-0.59 0.86	.72
Income	< = 5820	1. (Ref)			1. (Ref)			1. (Ref)			1. (Ref)			1. (Ref)			1. (Ref)		
	>5820	-0.28	-1.88 1.31	.73	0.20	-0.19 0.59	.31	0.17	-0.23 0.56	.41	0.32	-0.25 0.91	.27	0.33	-0.49 1.15	.43	0.31	-0.51 1.14	.46
Type leprosy	PB	1. (Ref)			1. (Ref)			1. (Ref)			1. (Ref)			1. (Ref)			1. (Ref)		
	MB	1.32	-0.66 3.32	.19	0.25	-0.47 0.97	.50	0.52	-0.20 1.24	.16	0.12	-0.47 0.72	.68	-0.27	-1.09 0.56	.53	-0.15	-0.97 0.68	.73
Distance to nearest facility	< = 6 km	1. (Ref)			1. (Ref)			1. (Ref)			1. (Ref)			1. (Ref)			1. (Ref)		
	>6 km	-1.49	-3.25 0.27	.10	-0.15	-0.63 0.33	.53	-0.22	-0.70 0.27	.38	-.44	-0.89 0.01	.06	-0.43	-1.07 0.22	.19	-0.47	-1.12 0.17	.15
Type of facility visited	Private	1. (Ref)			1. (Ref)			1. (Ref)			1. (Ref)			1. (Ref)			1. (Ref)		
	Both	-3.11	-3.64 -2.58	**.000**	-1.09	-2.49 0.31	.13	-1.89	-3.29 -0.48	**.01**	0.02	-0.62 0.66	.95	-0.79	-1.49 -0.09	**.03**	-1.04	-1.74 -0.35	**.00**
	Government	-2.81	-3.49 -2.13	**.000**	-0.94	-2.64 0.76	.28	-1.62	-3.31 0.08	.06	-0.91	-2.01 0.18	.10	-0.51	-1.50 0.48	.31	-0.35	-1.34 0.64	.49
OPD visits frequency	1	1. (Ref)			1. (Ref)			1. (Ref)			1. (Ref)			1. (Ref)			1. (Ref)		
	2	-2.44	-3.54 -1.35	**.000**	0.09	-1.32 1.49	.90	0.05	-1.36 1.45	.95	0.01	-0.47 0.50	.95	-0.26	-2.26 1.74	.80	-0.13	-2.13 1.87	.90
	3	-2.67	-4.64 -0.70	**.01**	0.27	-0.19 0.73	0.25	0.21	-0.25 0.67	.38	-0.31	-1.55 0.91	.61	-0.02	-0.73 0.69	.96	0.11	-0.60 0.82	.77
HH size	< = 5	1. (Ref)			1. (Ref)			1. (Ref)			1. (Ref)			1. (Ref)			1. (Ref)		
	>5	0.01	-1.57 1.59	.99	-0.29	-0.69 0.12	0.16	-0.30	-0.70 0.10	.14	0.11	-0.36 0.59	.63	-0.06	-0.73 0.60	.85	-0.12	-0.78 0.55	.73
Catastrophic exp.	No	1. (Ref)			1. (Ref)			1. (Ref)			1. (Ref)			1. (Ref)			1. (Ref)		
	Yes	2.92	1.86 3.98	**.000**	0.77	-0.05 1.60	0.07	1.21	0.39 2.03	**.00**	1.00	0.23 1.77	**.01**	1.25	-0.18 2.68	.09	1.29	-0.13 2.72	.08

Table 5 presents the association when only statistically significant variables (p < 0.05) are modelled together with total expenditure (direct + indirect). When modelled separately for both sites, all the variables in Umbergaon turned statistically not-significant. Age however, remained a statistically significant factor (p = 0.03) in DNH. The overall model (Omnibus Test) was statistically significant in DNH (p = 0.001), but not in Umbergaon (p = 0.06). Furthermore, the same model was applied jointly for DNH and Umbergaon (n = 140), which was overall highly significant (p ≤ 0.001). The age (p = 0.019) and type of facility (p = 0.002) were statistically significant, but catastrophic expenditure became statistically not-significant. Catastrophic coefficients however, indicated that catastrophic expenditure groups (in both the areas) had risk of spending (total expenditure) almost twice, compared to non-catastrophic groups.

**Table 5:** Socioeconomic factors associated with total expenditure by leprosy patients on outpatient services in DNH and Umbergaon (multivariate analysis).

		Total expenditure											
		DNH (N = 103)				Umbergaon (N = 37)				DNH+ Umbergaon (N = 140)			
		Coef.	Std Err.	95% CI	p	Coef.	Std Err.	95% CI	p	Coef.	Std Err.	95% CI	p
Age	< = 18	1. (Ref)				1. (Ref)				1. (Ref)			
	19–35	-0.31	0.27	-0.83 0.21	.25	0.06	0.49	-0.89 1.01	.91	-0.21	0.23	-0.66 0.23	.35
	> = 36	-0.53	0.23	-0.98 -0.08	**.02**	-0.20	0.49	-1.16 0.76	.69	-0.47	0.20	-0.86 -0.08	**.02**
Type of facility visited	Pvt.	1. (Ref)				1. (Ref)				1. (Ref)			
	Both	-0.67	1.02	-2.68 1.33	.51	-0.20	0.55	-1.28 0.88	.72	-0.24	0.42	-1.06 0.58	.56
	Gov.	-1.08	0.88	-2.80 0.64	.22	-0.80	0.44	-1.67 0.07	.07	-0.80	0.26	-1.30 -0.29	**.00**
Catastrophic exp.	No	1. (Ref)				1. (Ref)				1. (Ref)			
	Yes	0.58	0.51	-0.43 1.59	.26	0.97	0.77	-0.54 2.48	.21	0.73	0.39	-0.03 1.48	.06

## Discussion

Our study explored the leprosy patient’s financial burden due to primary care outpatient services. Primary care is an important aspect of disease control under a public health program, therefore costs at this level are important for policy and planning. Moreover, a high out of pocket expenditure indicates public health systems inefficiency, and act as barrier to access services [46]. The results show that the sampled patients were mainly in their economically productive lifetime, indicating leprosy imposing a high economic burden. The leprosy patients of DNH went more frequently to the OPD, and preferred a government facility as compared to Umbergaon. Furthermore, the total expenditure (direct + indirect) was statistically significantly lower in DNH than Umbergaon. The age of the leprosy patients and type of health facilities were the major predictors of total expenditure. The higher the age, the higher the expenditure, and private health facilities were more expensive than government facilities, at both sites.

As a limitation, our study only considered direct and indirect costs, however skin anesthesia (a common phenomenon), neuropathic pain [47, 48], poor mental health [49] and stigma [49, 50] can be significant factors, which can elevate the total expenditure further. We could not focus on these parameters under patient characteristics, and recommend to explore this in detail in future. Next, the households belong to poor socioeconomic groups, which correlates with other studies [9, 13, 22], but we drew the sample from government records, which often caters mainly to poor. Also, adequate representation of patients who are diagnosed and treated completely in private facilities cannot be ascertained. The relatively small sample size is also a limitation of this study. The sample size turned out to be low (reduced power) because of high zero visits, meaning that patients often did not visit the outpatient clinics according to the official schedule. Moreover, to minimize recall bias, we only included the patients of the most recent one year, which was a small cohort. Many patients were not traceable due to migration. Furthermore, we computed catastrophic expenditure based on the income, rather than consumption pattern, which is a more rigorous method. The study is cross-sectional and there is no insight on how patients adapt over time. We recommend to repeat the survey after an appropriate time gap. Also, OPD expenditure is not as high as hospitalization, therefore often failed to be recalled. We do not reject the possibility of recall bias, but we further reduced this by averaging the expenditure from last three visits. Although we have quantified health seeking behaviour, this study does not identify the underlying reasons for these patterns, which would further necessitate qualitative studies.

So far, sound evidence is lacking on the private sector uptake of leprosy cases, therefore we compared the patient’s selection of health facilities for primary leprosy care. We observed that the government is mostly preferred over private health facilities (government 80.8% *vs*. private 1.7%) in an enhanced health system (DNH). In a non-enhanced health system (Umbergaon) however, private is equally preferred (private 15% *vs*. government 11.7%). Moreover, in a non-enhanced health system (Umbergaon) patients have poor health seeking behaviour (zero OPD visits in last 6 months: Umbergaon 69% *vs*. DNH 14%). Contrary to the high number of subjects reporting zero visits, the predicted probability of zero direct medical expenditure (Umbergaon 0.35 *vs*. DNH 0.88) is lower in Umbergaon, and *vice versa* in DNH. It means that patients in Umbergaon avoid visiting any health facility, but if they visit then end up paying more than in DNH, therefore out of pocket direct medical expenditure acts as a potential barrier to access leprosy health care. The indirect expenditure is the largest cost impoverishing component for patients. Next, the indirect expenditure with transportation and total expenditure in an enhanced health system (DNH) is lower than non-enhanced health system (Umbergaon). Usually, a high variation is expected in indirect expenditure and transportation, because in many instances they are not paid out of pocket and are presumptive e.g. wage loss. This can lead to over or under reporting. For example, many people use their own vehicle or are supported by others, and often fail to report this. This in turn leads to unrealistic and non-comparable estimates, which are of low utility for policy purposes. Therefore, we used standard government labour market and transportation rates in both areas for comparable results, which are appropriate for the sampled socioeconomic groups. Our study identifies the linkage between socioeconomic factors and expenditure increase. The total expenditure peaked at the 19–35 age category, which correlates with the human capital approach, i.e. the productive age group is more weighted than early or old age [39, 51]. Next, private health facilities are significantly more expensive than government facilities, therefore one of the reasons for higher total expenditure in Umbergaon than DNH.

We conclude that the condition of public health systems has a direct relationship with the patient’s expenditure, and the better the public health system, the lesser the expenditure from the leprosy patient’s pocket. Next, the condition of public health system has a major effect on the patient’s health seeking behaviour, i.e. selection of health facility and services uptake. If a health system is weak, then leprosy patients are forced to seek private health care, which is more expensive and imposes a significant financial burden on the leprosy affected population, proven to be catastrophic. If a public health system is enhanced, then patients prefer to avail government health facility services. We recommend to invest in health system strengthening to reduce the economic burden of leprosy.

## Supporting information

S1 Checklist STROBE checklist. (DOCX)

S1 Dataset Data. (XLSX)

S1 Table Model comparison for direct and indirect expenditure on leprosy outpatient care. (DOCX)
