# Supplementary material for: Household expenditure on leprosy outpatient services in the Indian health system: A comparative study
Source: PLoS Negl Trop Dis. 2018 Jan 4;12(1):e0006181. doi: 10.1371/journal.pntd.0006181 (PMC5771634; doi:10.1371/journal.pntd.0006181)
Supplement: S1 Checklist — (DOCX) [file pntd.0006181.s001.docx]

STROBE Statement—Checklist of items that should be included in reports of ***observational studies***

|  | Item No | Recommendation |
| --- | --- | --- |
| **Title and abstract** | 1 | (*a*) Indicate the study’s design with a commonly used term in the title or the abstract  **[Within the title on page 1 and method section of the abstract page 2]** |
|  |  | (*b*) Provide in the abstract an informative and balanced summary of what was done and what was found**[ results section of the abstract page 2]** |
| Introduction | | |
| Background/rationale | 2 | Explain the scientific background and rationale for the investigation being reported  **[page 4 (send para)-5 (first para)]** |
| Objectives | 3 | State specific objectives, including any prespecified hypotheses  [**page 5 last paragraph]** |
| Methods | | |
| Study design | 4 | Present key elements of study design early in the paper **[Subheading study design page 6]** |
| Setting | 5 | Describe the setting, locations, and relevant dates, including periods of recruitment, exposure, follow-up, and data collection **[Subheading Study sites and Data Collection and analysis page 6-8]** |
| Participants | 6 | (*a*) Give the eligibility criteria, and the sources and methods of selection of participants. Describe methods of follow-up**[Subheading Study design page 6]** |
|  |  | (*b*) For matched studies, give matching criteria and number of exposed and unexposed **[N/A]** |
| Variables | 7 | Clearly define all outcomes, exposures, predictors, potential confounders, and effect modifiers. Give diagnostic criteria, if applicable **[N/A]** |
| Data sources/ measurement | 8* | For each variable of interest, give sources of data and details of methods of assessment (measurement). Describe comparability of assessment methods if there is more than one group **[N/A]** |
| Bias | 9 | Describe any efforts to address potential sources of bias **[Discussion page 20 (last para)-21 (first para)]** |
| Study size | 10 | Explain how the study size was arrived at **[Subheading Study design page 6]** |
| Quantitative variables | 11 | Explain how quantitative variables were handled in the analyses. If applicable, describe which groupings were chosen and why [**Subheading Data Collection and analysis (page 9-10) and Data modelling page (page 11)]** |
| Statistical methods | 12 | (*a*) Describe all statistical methods, including those used to control for confounding**[Subheading Data Collection and analysis; Data modelling page (page 11)]** |
|  |  | (*b*) Describe any methods used to examine subgroups and interactions **[N/A]** |
|  |  | (*c*) Explain how missing data were addressed**[Subheading Data Collection and analysis page 9 ]** |
|  |  | (*d*) If applicable, explain how loss to follow-up was addressed **[N/A]** |
|  |  | (*e*) Describe any sensitivity analyses**[N/A]** |
| Results | | |
| Participants | 13* | (a) Report numbers of individuals at each stage of study—eg numbers potentially eligible, examined for eligibility, confirmed eligible, included in the study, completing follow-up, and analysed **[Results sections page 12]** |
|  |  | (b) Give reasons for non-participation at each stage **[Discussion page 20]** |
|  |  | (c) Consider use of a flow diagram **[N/A]** |
| Descriptive data | 14* | (a) Give characteristics of study participants (eg demographic, clinical, social) and information on exposures and potential confounders **[page 8]** |
|  |  | (b) Indicate number of participants with missing data for each variable of interest **[Results page 13 table]** |
|  |  | (c) Summarise follow-up time (eg, average and total amount) **[N/A]** |
| Outcome data | 15* | Report numbers of outcome events or summary measures over time**[N/A]** |
| Main results | 16 | (*a*) Give unadjusted estimates and, if applicable, confounder-adjusted estimates and their precision (eg, 95% confidence interval). Make clear which confounders were adjusted for and why they were included **[N/A]** |
|  |  | (*b*) Report category boundaries when continuous variables were categorized **[Results Table 3, 4, 5]** |
|  |  | (*c*) If relevant, consider translating estimates of relative risk into absolute risk for a meaningful time period **[N/A]** |
| Other analyses | 17 | Report other analyses done—eg analyses of subgroups and interactions, and sensitivity analyses **[N/A]** |
| Discussion | | |
| Key results | 18 | Summarise key results with reference to study objectives **[Discussion page 20 first para]** |
| Limitations | 19 | Discuss limitations of the study, taking into account sources of potential bias or imprecision. Discuss both direction and magnitude of any potential bias**[Discussion page 20 last para]** |
| Interpretation | 20 | Give a cautious overall interpretation of results considering objectives, limitations, multiplicity of analyses, results from similar studies, and other relevant evidence**[Discussion page 20-22]** |
| Generalisability | 21 | Discuss the generalisability (external validity) of the study results **[N/A]** |
| Other information | | |
| Funding | 22 | Give the source of funding and the role of the funders for the present study and, if applicable, for the original study on which the present article is based**[Financial Disclosure]** |

*Give information separately for exposed and unexposed groups.

**Note:** An Explanation and Elaboration article discusses each checklist item and gives methodological background and published examples of transparent reporting. The STROBE checklist is best used in conjunction with this article (freely available on the Web sites of PLoS Medicine at http://www.plosmedicine.org/, Annals of Internal Medicine at http://www.annals.org/, and Epidemiology at http://www.epidem.com/). Information on the STROBE Initiative is available at http://www.strobe-statement.org.
