# Supplementary material for: Household expenditure on leprosy outpatient services in the Indian health system: A comparative study
Source: PLoS Negl Trop Dis. 2018 Jan 4;12(1):e0006181. doi: 10.1371/journal.pntd.0006181 (PMC5771634; doi:10.1371/journal.pntd.0006181)
Supplement: S1 Table — (DOCX) [file pntd.0006181.s003.docx]

**S1 Table. Model comparison for direct and indirect expenditure on leprosy outpatient care**

**S1.1. Model comparison of direct expenditures on leprosy outpatient care (supporting table 3 in the manuscript)**

| **Dependent var.** | **Independent var.** | **Zero inflated var.** | **zip*** | | **vuong**** | |
| --- | --- | --- | --- | --- | --- | --- |
|  |  |  | **chibar2** | **Pr>=chibar2** | **z** | **Pr>z** |
| Consultation | Patient area | Type of facility and OPD frequency | 1530.34 | 0.0000 | 7.86 | 0.0000 |
| Medicines & supplies | Patient area | Type of facility and OPD frequency | 1.1e+04 | 0.0000 | 5.32 | 0.0000 |
| Direct medical expenditure | Patient area | Type of facility and OPD frequency | 1.4e+04 | 0.0000 | 4.72 | 0.0000 |

*zero inflated negative binomial vs. zero inflated poisson

**zero inflated negative binomial vs. standard negative binomial

**S1.2. Model comparison of transportation and indirect expenditures on leprosy outpatient care (supporting table 3 in the manuscript)**

| **Dependent var.** | **Independent var.** | **Poisson** | | **Negative binomial** | |
| --- | --- | --- | --- | --- | --- |
|  |  | **Deviance** | **AIC** | **Deviance** | **AIC** |
| Transportation | Patient area | 24.748 | 4226.63 | 0.419 | 1444.5 |
| Patient's wageloss (assumed all adults) | Patient area | 209.224 | 29936.564 | 0.418 | 1946.839 |
| Attendant’s wage loss | Patient area | 153.188 | 21885.321 | 3.234 | 1765.848 |
| Transport+Indirect exp. (assumed all adult) | Patient area | 195.994 | 28193.507 | 0.284 | 2093.485 |
| Total (direct+ indirect) exp. (assumed all adults) | Patient area | 318.302 | 45083.586 | 0.381 | 2129.936 |

**S1.3. Model comparison of socioeconomic factors associated with direct medical expenditures on outpatient services in DNH and Umbergaon (supporting table 4 in the manuscript)**

| **Dependent var.** | **Independent var.** | **zero inflated var.** | **Umbergaon** | | | | | | **DNH** | | | | | |
| --- | --- | --- | --- | --- | --- | --- | --- | --- | --- | --- | --- | --- | --- | --- |
|  |  |  | **Overall model** | | **zip*** | | **vuong**** | | **Overall model** | | **zip*** | | **vuong**** | |
|  |  |  | **LR chi2** | **Prob > chi2** | **chibar2** | **Pr>=chibar2** | **z** | **Pr>z** | **LR chi2** | **Prob > chi2** | **chibar2** | **Pr>=chibar2** | **z** | **Pr>z** |
| Direct medical expenditure per visit | Age | Type of facility and OPD frequency | 0.46 | 0.7954 | 3603.55 | 0.0000 | 2.36 | 0.0092 | 11.50 | 0.0032 | 5249.57 | 0.0000 | 3.10 | 0.0010 |
|  | Sex | Type of facility and OPD frequency | 1.83 | 0.1761 | 3418.71 | 0.0000 | 2.59 | 0.0048 | 0.29 | 0.5883 | 1.0e+04 | 0.0000 | 3.07 | 0.0011 |
|  | Occupation | Type of facility and OPD frequency | 2.29 | 0.1306 | 3381.50 | 0.0000 | 2.19 | 0.0144 | 2.56 | 0.1096 | 9223.60 | 0.0000 | 3.31 | 0.0005 |
|  | Income | Type of facility | 1.11 | 0.2929 | 3524.95 | 0.0000 | 2.55 | 0.0053 | 0.12 | 0.7275 | 1.0e+04 | 0.0000 | 2.7 | 0.0035 |
|  | Type leprosy | Type of facility and OPD frequency | 0.17 | 0.6766 | 3637.75 | 0.0000 | 2.55 | 0.0054 | 2.08 | 0.1492 | 8379.49 | 0.0000 | 2.90 | 0.0019 |
|  | Distance to nearest facility | OPD frequency | 3.32 | 0.0682 | 3251.21 | 0.0000 | 1.94 | 0.0259 | 1.89 | 0.1688 | 9168.17 | 0.0000 | 2.67 | 0.0038 |
|  | Type of facility | OPD frequency | 3.42 | 0.1806 | 3328.78 | 0 | 1.88 | 0.3 | 19.79 | 0.0001 | 554.99 | 0.0000 | 2.39 | 0.0084 |
|  | OPD frequency | Type of facility | 0.25 | 0.8827 | 3631.33 | 0.0000 | 2.66 | 0.0040 | 13.09 | 0.0014 | 4225.98 | 0.0000 | 2.49 | 0.0065 |
|  | HH size | Type of facility and OPD frequency | 0.23 | 0.6315 | 3630.86 | 0.0000 | 2.41 | 0.0079 | 0.00 | 0.9854 | 1.0e+04 | 0.0000 | 3.13 | 0.0009 |
|  | Catastrophic expenditure | OPD frequency | 7.52 | 0.0061 | 2521.74 | 0.0000 | 1.30 | 0.0968 | 19.35 | 0.0000 | 577.88 | 0.0000 | 2.48 | 0.0066 |

*zero inflated negative binomial vs. zero inflated poisson

**zero inflated negative binomial vs. standard negative binomial

**S1.4. Model comparison of socioeconomic factors associated with transport + indirect expenditures on outpatient services in DNH and Umbergaon (supporting table 4 in the manuscript)**

| **Dependent var.** | **Independent var.** | **Umbergaon** | | | | **DNH** | | | |
| --- | --- | --- | --- | --- | --- | --- | --- | --- | --- |
|  |  | **Poisson** | | **Negative binomial** | | **Poisson** | | **Negative binomial** | |
|  |  | **Deviance** | **AIC** | **Deviance** | **AIC** | **Deviance** | **AIC** | **Deviance** | **AIC** |
| Transport+ Indirect expenditure per visit | Age | 201.058 | 7153 | 0.215 | 573.3 | 130.664 | 13903.7 | 0.214 | 1517.6 |
|  | Sex | 275.796 | 9967.9 | 0.305 | 574.7 | 165.884 | 17589.6 | 0.274 | 1521.9 |
|  | Occupation | 286.690 | 10349.2 | 0.318 | 575.1 | 158.195 | 16813.1 | 0.260 | 1520.4 |
|  | Income | 276.592 | 9995.7 | 0.304 | 574.7 | 161.788 | 17175.9 | 0.267 | 1521.1 |
|  | Type leprosy | 280.907 | 10146.8 | 0.310 | 574.9 | 164.468 | 17446.6 | 0.272 | 1521.7 |
|  | Distance to nearest facility | 250.367 | 9077.9 | 0.273 | 573.6 | 165.392 | 17540.0 | 0.273 | 1521.8 |
|  | Type of facility | 184.304 | 6583.4 | 0.186 | 572.4 | 141.206 | 14958.0 | 0.245 | 1520.7 |
|  | OPD frequency | 281.265 | 10438.6 | 0.328 | 577.2 | 165.898 | 17754.9 | 0.266 | 1522.8 |
|  | HH size | 288.446 | 10410.6 | 0.320 | 575.3 | 155.765 | 16567.6 | 0.257 | 1520.2 |
|  | Catastrophic expenditure | 146.385 | 5438.5 | 0.197 | 571.0 | 137.143 | 14686.7 | 0.235 | 1517.9 |
